# Evaluation of a novel role proposal for the use of a physiotherapist navigator in an acute cancer care setting in Ontario: Protocol for a pilot randomized controlled trial

**DOI:** 10.1371/journal.pone.0351761

**Published:** 2026-06-23

**Authors:** Holly Edward, Luciana Macedo, Sarah Wojkowski, Som D. Mukherjee, Jenna Smith-Turchyn

**Affiliations:** 1 School of Rehabilitation Science, McMaster University, Hamilton, Ontario, Canada; 2 Oncology, McMaster University, Hamilton, Ontario, Canada; Institute of Public Health from Guanajuato State, MEXICO

## Abstract

**Background:**

An increasing number of Canadians are living beyond cancer, with many individuals experiencing long-lasting side effects of cancer and its treatments. Research shows that engaging in rehabilitation during and after cancer treatment can reduce side effects and improve quality of life. However, less than 30% of individuals with cancer have access to rehabilitation services. The purpose of this pilot trial is to assess the feasibility of a novel PT Navigator role in an acute cancer care setting for individuals undergoing treatment for cancer.

**Methods:**

*Study Design:* Pilot randomized controlled trial. *Eligibility:* Adults aged ≥18 years who are currently undergoing cancer treatment for any type of cancer. *Intervention:* The intervention group includes six consultative sessions by a PT Navigator over a maximum of eighteen weeks. *Randomization:* Participants will be randomly allocated using a 1:1 allocation ratio to receive the intervention with the PT Navigator or usual care. *Outcomes:* The primary feasibility outcome is adherence rate. Secondary outcomes include recruitment rate, retention rate, satisfaction and preliminary effectiveness, including health-related quality of life, self-reported exercise volume, health care utilization, activity limitation, functional strength, exercise capacity, and overall impairment score. *Analysis:* Outcomes will be assessed at baseline, post-intervention, and two month follow up. Feasibility and preliminary effectiveness will be assessed using descriptive statistics.

**Discussion:**

This study aims to assess the feasibility of a novel PT Navigator role in an acute cancer care setting in Ontario, Canada. This pilot trial will evaluate process and resource capabilities before testing the role within a larger scale randomized controlled trial. The overall project goal is to facilitate regular assessment by a rehabilitation professional to promote early identification of physical impairment and early intervention to manage impairment as a standard component of cancer care in Canada.

**Trial registration:**

ClinicalTrials.gov ID NCT07045740.

## Introduction

The burden of cancer in Canada is growing with nearly one in two [1], or 43% [2], of Canadians being diagnosed with cancer throughout their lifetime. With survival rates increasing [2,3], individuals living beyond cancer face many persistent symptoms that can affect their day-to-day functioning and overall quality of life [4–6]. Cancer and cancer treatments can lead to significant adverse effects such as pain, fatigue, lymphedema and reduced mobility. Research demonstrates that engaging in physical activity and rehabilitation can enhance quality of life, improve physical function, and reduce anxiety and depression amongst those living with and beyond cancer [7,8]. Additionally, recently published research involving patients with colorectal cancer has shown that engaging in physical activity and exercise during and after cancer treatment reduced the risk of cancer recurrence and resulted in improved overall survival [9]. Physiotherapists (PTs) are specialists in physical rehabilitation and health promotion in Canada [10]. Current provincial [11] and international [12,13] oncology clinical practice guidelines recommend that PTs are included as part of the cancer care team across all stages of cancer treatment. However, only 30% of cancer survivors in Canada have access to rehabilitation [14] leaving individuals to find and fund their own rehabilitation, something many are unable to do [15,16].

There is a need to identify gaps in service and challenges to accessing care when considering rehabilitation for managing long-term impairments [17]. In 2012, Stout et al. developed a Prospective Surveillance Model (PSM) for rehabilitation for women with breast cancer [18]. The goal of the PSM is to facilitate early identification of impairments through ongoing surveillance throughout all stages of cancer care [18]. Current literature examining the application of the PSM across several countries has demonstrated cost-effectiveness [19–21] and provided evidence that proactive monitoring and early intervention can prevent side effects [20,22,23]. In addition to the PSM, healthcare navigator roles have been shown to reduce barriers to accessing treatment [17,24], enhance care processes [17,24], and reduce clinical costs across healthcare settings [24,25].

Canadians living with cancer have expressed an unmet need and want to participate in physical activity, exercise, and rehabilitation to alleviate long term side effects of cancer, facilitate their return to work, and increase quality of life [26–28]. Navigators frequently collaborate with patients and their families to provide knowledge, support, and guidance on local health care systems or community services available to them [29]. A scoping review exploring PTs in a navigator role found that navigation in cancer treatment settings has increasingly emerged over the last ten years, however, currently most navigator positions in cancer care settings are held by nurses and nearly all PT Navigator roles are from the United States of America [30]. The review further highlighted how a PT Navigator could effectively identify and address common patient reported barriers and facilitate awareness on the benefits of rehabilitation across the cancer care continuum [30]. Moreover, the role would allow symptom assessment and early management to be a part of standard and routine care [16,30]. Despite the known benefits of implementing prospective monitoring and patient navigation, no study evaluating the use of a PT Navigator has been conducted in Canada.

## Methods

### Study purpose

The purpose of this pilot RCT is to examine the feasibility of a PT Navigator role for individuals living with cancer receiving cancer treatment in an acute cancer care setting. Specifically, our research questions are as follows:

*Research question 1 (Primary):* Is a PT-Navigator role feasible, as measured by adherence, recruitment, retention, and satisfaction rates, to implement at a cancer institution in Ontario, Canada?

*Research question 2 (Secondary):* Do individuals with cancer who are monitored by a PT-Navigator demonstrate trends for improved outcomes in overall impairment score, health-related quality of life, physical activity levels, exercise capacity, functional strength, activity limitation, and a reduction in health care utilization costs compared to individuals with cancer who are not monitored by a PT Navigator during treatment?

### Study design

**Fig 1:**
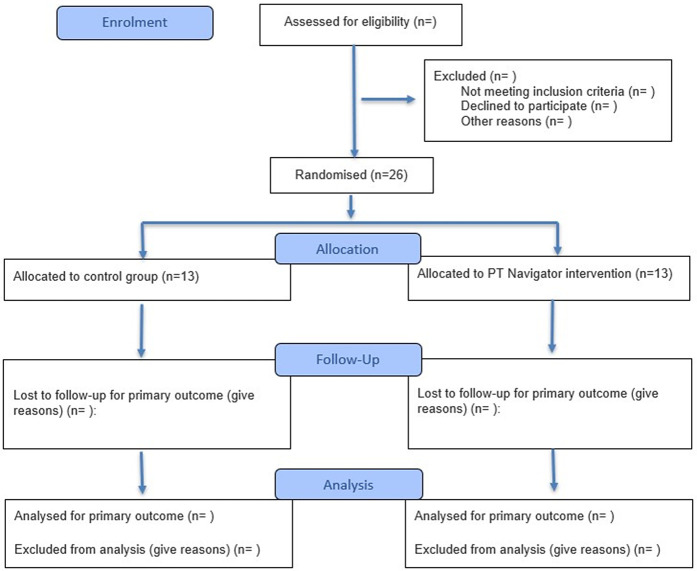
Study Flow Chart.

**Fig 2:**
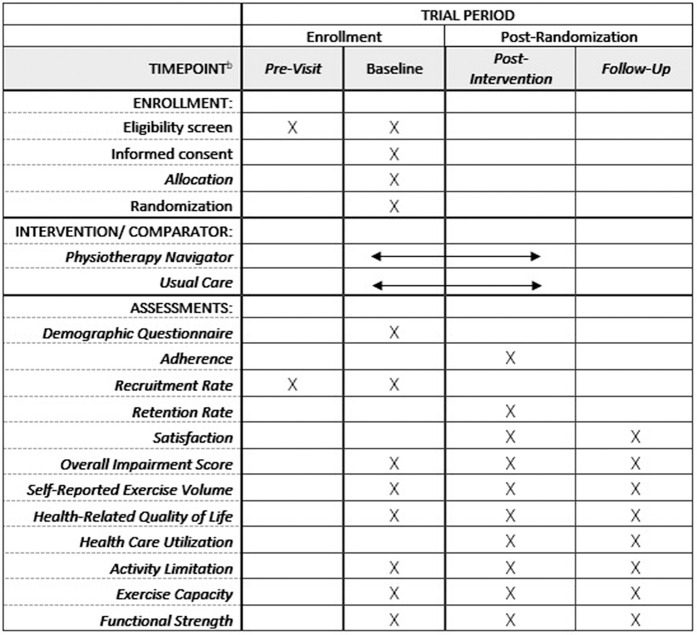
Schedule of Enrollment, Interventions, and Assessments (SPIRIT Statement).

This is a two-arm pilot randomized controlled trial [31] (Figs 1 and 2). This protocol is reported according to the CONSORT guidelines extended to pilot and feasibility trials [32] and the SPIRIT guidelines for protocols of randomized trials [33]. See S1 Appendix for the SPIRIT 2025 checklist of items to address in a randomized trial protocol. The Hamilton Integrated Research Ethics Board has reviewed and approved this study (#18583). The PT Navigator role was developed and examined first using a scoping review [30] to explore the current design and utility of a PT in a navigational role and further developed to a Canadian context using a modified Delphi study [34] seeking the feedback of a role proposal through various end-users across Canada (e.g., PTs, oncologists, nurses). See S2 Appendix for the finalized role description.

### Recruitment

All eligible individuals living with cancer who attend the Juravinski Cancer Centre (JCC), a regional cancer care centre in Hamilton, Ontario, between November 2025 and February 2026, will be approached consecutively by their medical oncologist to participate in the study. The medical oncologists will connect the potential participant to the research team (HE) who will confirm eligibility and start the consent process prior to enrollment. Consent will be obtained both verbally and using written documentation through ethics-approved consent forms, further witnessed by a member of the research team (HE). There will also be the opportunity for self-referral as recruitment flyers will be advertised within the cancer centre for patients to contact the study team directly. A rolling recruitment and enrollment strategy will be used until the required sample size is achieved. Participants allocated to the intervention group will be introduced by their medical oncologist to the PT Navigator early during their cancer treatment (i.e., within the first month).

### Participants

Eligible participants will include adults aged ≥18 years old, who have been recently diagnosed with any type/stage of cancer who are/will be receiving cancer treatment (e.g., chemotherapy, radiation, immunotherapy), are English-speaking, and are community-dwelling. Participants will be excluded from the study if they have a self-reported physical or cognitive impairment that would prevent them from carrying out a physical assessment.

### Sample size

Our sample size was calculated using a confidence interval approach based on recommendations for pilot trials [35–37]. Specifically, we used a 95% confidence interval, a desired margin of error of 0.2, and our estimated 70% adherence rate to determine feasibility. The calculated sample size was 20 participants. However, to account for a 20% dropout rate, the final sample size will include 13 participants per group (26 total). It is important to consider that as the pilot study is exploratory in nature, this sample size is not powered to detect meaningful change between groups.

### Randomization and allocation

Randomization will be completed by a member of the research team using RedCap [38]. The 1:1 randomization schedule will be generated using permuted blocks by a member of the research team not involved in recruitment, randomization or treatment delivery. Participants allocated to the intervention group will receive a phone call by the PT Navigator to schedule the first intervention session.

### Blinding

Due to the nature of the intervention, the participants and PT Navigator cannot be blinded to group allocation. All data will be de-identified for data analysis by a blinded statistician.

### Procedure

**Fig 3:**
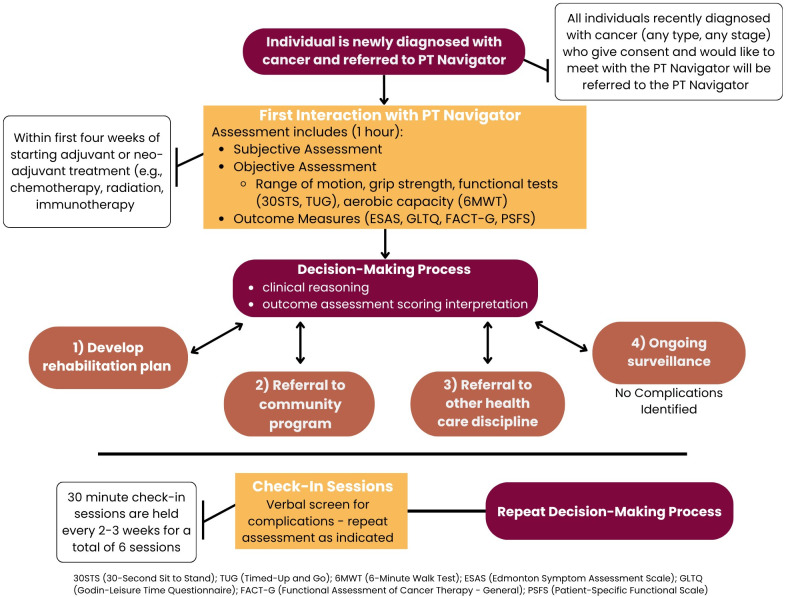
PT Navigator Role Process.

Intervention Group (PT Navigator). The interactions with the PT Navigator will occur at the JCC. The PT Navigator will follow the PSM [18] for ongoing monitoring and surveillance. The PT Navigator will check-in with participants approximately every two to three weeks over a maximum of 18 weeks (i.e., six sessions total with the PT Navigator) when they are already coming to the JCC for another appointment. Interactions with the PT Navigator will be 30 minutes (follow-ups) to one hour (initial assessment). Fig 3 represents the PT Navigator interaction process.

The PT Navigator will administer outcome measures and continuously screen for complications or side effects of treatment (i.e., fatigue, chemotherapy-induced peripheral neuropathy). See Table 1 for a summary of outcome measures and scoring interpretations to guide the PT Navigator in their decision-making on next steps. However, the PT Navigator will always use their clinical reasoning to determine what clinical tests they will assess and to guide their decision making. The PT Navigator will triage participants with one or more of the following steps based on their assessment: 1) Develop a rehabilitation plan (i.e., discuss self-management strategies, exercise prescription, goal setting, rehabilitation barrier identification), 2) Refer the individual to a community program (i.e., the patient needs more frequent follow-up and/or more supervision with rehabilitation services), 3) Refer to another health care discipline (i.e., the patient needs care outside of the PT scope of practice – for example, to discuss changes in weight and appetite with a dietician), 4) Continue with ongoing surveillance (i.e., no complications of treatment identified – continue to provide positive reassurance, answer questions, and provide education on maintenance strategies). Note, patient consent will be received throughout all interactions and triaging decisions with the PT Navigator, including regarding any communication and documentation which will follow the Documentation Standard as set out by the College of Physiotherapists of Ontario [39] and the Personal Health Information Protection Act [40].

**Table 1:** Outcome Measures and Decision-Making Process.

Outcome(s) Assessed and Measure Used	Scoring Interpretation	Decision-Making Process
**Overall Impairment** Edmonton Symptom Assessment Scale (ESAS)	0 = none 1–3 = mild 4–6 = moderate 7–10 = severe -symptoms are assessed on a 0–10 scale with 10 representing worst severity -minimal clinically important difference = 2 points	-**mild** = PT Navigator to provide education on monitoring and how to prevent symptom worsening -**moderate/severe** = PT Navigator continues monitoring, communicate symptoms with medical team, and prescribe/deliver intervention or referral as appropriate
**Self-Reported Exercise Volume** Godin Leisure-Time Questionnaire	24 units or more = active 14-23 units = moderately active less than 14 units = insufficiently active/sedentary -minimal clinically important difference = 10% change in physical activity from baseline	-**active** = PT Navigator provides support and encouragement -**moderately active** = PT Navigator provides physical activity education and goal setting -**sedentary** = PT Navigator identifies barriers with patient, goal setting, and prescribe/deliver exercise intervention
**Health-Related Quality of Life** Functional Assessment of Cancer Therapy – General (Fact-G)	Total score: 0–108 -physical (0–28) -social (0–28) -emotional (0–24) -functional (0–28) -items are assessed from 0 to 4 with higher scores indicating better perceived QOL -minimal clinically important difference = 4–7 points	-**total score >90** = PT Navigator continues monitoring and providing support and education where appropriate -**total score 50–89** = PT Navigator continues monitoring, communicate concerns with medical team, and prescribe/deliver intervention or referral as appropriate -**total score <49** = PT Navigator informs medical team immediately for follow up, prescribe/deliver intervention or referral as appropriate
**Activity Limitation** Patient-Specific Functional Scale (PSFS)	- score of 0 indicates the activity cannot be performed, while a score of 10 indicates the activity can be performed at the same level as before their problem -minimal clinically important difference not established in cancer	-a **decrease in 2 points** in an activity score – the PT Navigator to prescribe/deliver an exercise intervention or refer to community physiotherapy as appropriate (i.e., the patient needs frequent follow up)
**Range of Motion** **Muscle Strength** Goniometry and Grip Strength (Dynamometer)	-range of motion normative values vary per joint being examined -to compare grip strength to normative values of the general population and if applicable, normative values of patients with advanced cancer -minimal clinically important difference not established in cancer	-a **decrease of 10 degrees** in range of motion and/or if grip strength is **less than the normative value** for the patient’s age group and/or **there is a reduction of 5 kg since baseline** – the PT Navigator to prescribe/deliver an exercise intervention or refer to community physiotherapy as appropriate (i.e., the patient needs frequent follow up)
**Functional Lower Extremity Strength** 30-Second Sit to Stand	-to compare total repetitions completed to normative values of the general population with respect to patient’s age group -minimal clinically important difference not established in cancer	-if score is **less than the normative value** for the patient’s age group and/or there is a **decrease in 2 repetitions since baseline** – the PT Navigator will prescribe/deliver an exercise intervention or refer to community physiotherapy as appropriate (i.e., the patient needs frequent follow up)
**Mobility** Timed Up and Go	-to compare time to normative values of the general population with respect to patient’s age group -minimal clinically important difference not established in cancer	-if score is **less than the normative value** for the patient’s age group and/or there is **an increase in 3 seconds since baseline** – the PT Navigator will prescribe/deliver an exercise intervention or refer to community physiotherapy as appropriate (i.e., the patient needs frequent follow up)
**Aerobic Capacity** 6-Minute Walk Test	-to compare distance walked to normative values of the general population with respect to patient’s age group -minimal clinically important difference = 22–66 metres	-if walked distance is **less than the normative value** for the patient’s age group and/or there is a **decrease in 42 metres since baseline** – the PT Navigator will prescribe/deliver an exercise intervention or refer to community physiotherapy as appropriate (i.e., the patient needs frequent follow up)

*Minimal clinically important difference (MCID) is the minimum difference that the patient can recognize as beneficial.

**Note: this table serves as a reference guide. The PT Navigator can administer other outcome measures as appropriate and will always follow their clinical training and reasoning when considering decision-making. References to support scoring interpretation and triaging decisions can be found in the finalized role description found in S2 Appendix.

The PT Navigator will also assist participants in setting goals and developing action plans focused on functional activity or reduction in symptoms during each visit. Importantly, the PT Navigator will serve as an advocate to ensure the patient feels supported and aware of all resources available to them. The participants allocated to the intervention group will also be given the PT Navigator’s contact information and will be encouraged to communicate with them if they ever have any questions or concerns throughout the intervention period.

Control (Usual Care). Participants in this group will have no interaction with the PT Navigator. They will continue with usual care regarding rehabilitation from their medical oncologist and oncology care team. This ranges from no discussion of rehabilitation to general advice to stay physically active [41]. We will track any interventions received outside of the hospital by all participants (e.g., visits with family doctor, community PT) using the health care utilization questionnaire included as a study outcome.

### Outcomes

As this study is a pilot RCT, the primary outcome is feasibility as measured by adherence (primary), retention, and recruitment rates. Satisfaction with the PT Navigator role will also be used to assess feasibility. Secondary estimates of effect will be measured for health-related quality of life (HRQOL), self-reported exercise volume, health care utilization, activity limitation, exercise capacity, functional strength and overall impairment score. Outcome data will be assessed and collected using REDCap [38] at three timepoints: baseline (T1), post-intervention (T2: 18 weeks), and 2-month follow up (T3). See Fig 2 for the assessment schedule. It will take approximately 45 minutes to complete the required outcome measures. Following consent, participants will complete a baseline demographic questionnaire. The demographic questionnaire will include the Gender Related Attributes Survey (GERAS) [42], cancer characteristics, and prior experience with a PT.

### Feasibility

We selected feasibility outcomes to determine if any modifications would be needed prior to conducting a larger scale trial. A “traffic light” approach [43] was used as criteria for demonstrating feasibility, see Table 2. A “green light” indicates moving forward with no changes, a “yellow light” indicates proceeding with some modifications, and a “red light” indicates that the outcome was not feasible without making significant modifications to our methods [43]. The primary outcome of this study is feasibility as measured by adherence rate [37,44,45]. Criteria for feasibility and threshold details for each outcome are outlined in Table 2. Success thresholds were determined based on prior studies in this field for adherence [46–49], retention [46,47], recruitment [46,50], and a priori by authors for satisfaction rates.

**Table 2:** Traffic Light Criteria.

Criteria	Traffic Light Criteria		
	Green Light	Yellow Light	Red Light
***Adherence***			
Proportion of sessions attended with the PT Navigator	>70% of sessions with the PT Navigator were attended by the participants	50-70% of sessions with the PT Navigator were attended by the participants	<50% of sessions with the PT Navigator were attended by the participants
***Retention***			
Proportion of enrolled participants who complete post-intervention and follow-up assessments	>70% of participants complete post-intervention and follow up assessments	50-70% of participants complete post-intervention and follow up assessments	<50% of participants complete post-intervention and follow up assessments
***Recruitment***			
Proportion of eligible patients who enrolled into the study	>70% of eligible participants enrolled into the study	50-70% of eligible participants enrolled into the study	<50% of eligible participants enrolled into the study
***Satisfaction***			
Proportion of participants who report satisfaction with the PT Navigator role	>75% of participants select 5–7 on a 7-point Likert scale (higher scores indicate greater satisfaction)	50-75% of participants select 5–7 on a 7-point Likert scale (higher scores indicate greater satisfaction)	<50% of participants select 5–7 on a 7-point Likert scale (higher scores indicate greater satisfaction)

### Secondary Effectiveness

***Overall Impairment Score:*** The Edmonton Symptom Assessment System (ESAS) [51] will be used to assess overall impairment. The ESAS is a valid, reliable, and responsive measure in assessing various symptoms in individuals living with cancer [52–54]. Symptoms assessed through the ESAS has evolved to encompass many common cancer-related symptoms including nausea, depression, anxiety, pain, tiredness, drowsiness, shortness of breath, appetite, and well-being [55]. Respondents will be asked to rate their symptoms on a visual analog scale ranging from 0 (i.e., no symptoms) to 10 (i.e., maximal symptoms) [51]. Higher scores represent higher perceived symptom burden [51].

***Self-Reported Exercise Volume:*** The Godin Leisure Time Exercise Questionnaire [56] will be used to assess self-reported exercise volume. This questionnaire asks respondents to classify the amount of mild, moderate, and strenuous weekly activity [56]. It provides a total weekly leisure activity score [56]. Published literature has found the questionnaire to be reliable in classifying individuals living with and beyond cancer as ‘active’ and ‘insufficiently active’ [56–58]. Higher scores represent higher weekly levels of exercise [56].

***HRQOL:*** To measure HRQOL, both the EORTC QLQ-C30 [59] and the Functional Assessment of Cancer Therapy – General (FACT-G) [60] will be used. The FACT-G is a 27 item self-administered questionnaire that measures four domains of HRQOL including physical, emotional, social, and functional wellbeing [61]. The EORTC QLQ-C30 is also a self-administered measure that incorporates five functional scales (physical, social, emotional, role, and cognitive), three symptom scales (pain, fatigue, and nausea/vomiting), global; health status, other common cancer-related symptoms including loss of appetite and insomnia, and perceived financial impact of the disease [62,63]. Both questionnaires are commonly used around the world for individuals living with and beyond cancer, and psychometric evidence does not generally recommend one questionnaire over the other as both demonstrate good reliability, validity, and responsiveness [64].

***Health Care Utilization:*** A piloted healthcare resources survey [65] will be completed by participants in both groups at T2 and T3. This questionnaire assesses loss of work, procedures received, and all health-related visits (i.e., family doctor, support services). All costs will be calculated according to current (2025) Ontario healthcare standards and presented in CAD [65].

***Activity Limitation:*** To quantify activity limitation, the *Patient-Specific Functional Scale (*PSFS) [66] will be used. The PSFS asks respondents to identify three activities they are unable to do or having difficulty with completing [66]. Published literature has found this questionnaire to be a valid, reliable, and responsive outcome measure for use in musculoskeletal conditions [66,67]. Higher scores indicate greater ability to perform the activity at the same level prior to living with cancer [66].

***Exercise Capacity:*** To measure exercise capacity, 6-minute walk test [68] will be used. This performance-based test measures the distance a person can walk in six minutes. This test has been found to be reliable and valid for individuals living with and beyond cancer [69]. Higher distances indicate greater exercise capacity and endurance [68].

***Functional Strength:*** To measure functional strength, the 30 second sit to stand test [70] will be used. The test measures how many times a person can sit-stand-sit in 30 seconds. This test has been found to be valid and reliable in individuals living with cancer [71,72]. Higher repetitions indicate greater functional/lower extremity strength [73].

### Analysis

Stata Statistical Software (Version 19) will be used for all statistical analyses [74]. Descriptive statistics including means and standard deviations will be calculated for continuous outcomes. If not normally distributed, medians and quartiles will be reported. Categorical data will be described through absolute and relative frequencies.

Our primary outcome of feasibility will be analyzed using descriptive statistics. As a pilot RCT, we are not powered to accurately assess the effectiveness of the PT Navigator on the specified effectiveness outcomes (i.e., overall impairment score, HRQOL, self-reported exercise volume, activity limitation, exercise capacity, functional strength). Descriptive statistics including means and 95% confidence intervals will be reported to observe trends in all outcomes. We will follow the CONSORT reporting guidelines for extension to pilot trials [32] when reporting our results.

### Oversight and monitoring

#### Trial and data monitoring.

The primary investigator (HE) and local principal investigator (JST) will meet weekly to monitor study processes and progress. A steering committee will meet every four months to review aspects of study implementation, including recruitment, data monitoring, and provide recommendations to address concerns and ensure intervention fidelity. Members will include the project lead and co-investigators. Between meetings, regular email communication will ensure investigators are aware of project progress.

#### Adverse event reporting and harms.

The number of adverse events including minor injuries (e.g., strain or minor scratch) and oncological-related emergencies (e.g., changes in blood count levels, treatment complications) [7,75] will be recorded and the primary investigator notified.

#### Dissemination plans.

The results of this study will be published in peer-reviewed journals, presented at relevant conferences and disseminated to clinicians and participants, as appropriate.

## Discussion

This study introduces a unique approach to integrating PTs early into cancer treatment for individuals recently diagnosed with cancer. The findings of this pilot RCT will help to determine important feasibility considerations that can be used to design a future larger scaled RCT. The pilot study will also provide insight on the trends of effect of the PT Navigator on overall impairment score, QOL, physical activity levels, exercise capacity, functional strength, activity limitation, and health care utilization costs. We hope that our findings may later improve how physiotherapy services are offered to individuals living with cancer and advocate for routine symptom assessment and monitoring to be a standard part of cancer care in Canada.

The risks involved in participating in this study are minimal. Rehabilitation during and after cancer treatment is safe and endorsed by leading provincial [11] and international organizations [12,13]. The PT Navigator running the intervention will be a registered PT who has extensive training on how to safely implement exercise for patients living with cancer. However, there is always a risk of adverse events occurring including minor injuries and oncological-related emergencies [7,75] with active interventions. Overall, the potential benefits of engaging with a PT Navigator aiming to increase rehabilitation, far outweigh the risks of minor injury.

### Limitations

The results from this study should be interpreted with an understanding of its limitations to the generalizability of the results. For example, the results of this pilot RCT will only be relevant when considering implementation of the larger trial to sites that are comparable to the JCC. Also, due to the nature of the intervention and project constraints, there will be minimal blinding, which can introduce bias. The research team will need to take these factors into consideration, including resource needs, funding, and management supports when implementing the larger trial.

### Trial status

Protocol Version Number: 3. Date: November 30, 2025. Recruitment start date: November 21, 2025. Approximate recruitment end date: February 2026. Any trial modifications will be updated in a timely manner on ClinicalTrials.gov and sent via email to appropriate parties.

## Supporting information

S1 Appendix SPIRIT 2025 checklist of items to address in a randomized trial protocol. (DOCX)

S2 Appendix PT Navigator Role Description. (DOCX)
