# Supplementary material for: Evaluation of a novel role proposal for the use of a physiotherapist navigator in an acute cancer care setting in Ontario: Protocol for a pilot randomized controlled trial
Source: PLoS One. 2026 Jun 23;21(6):e0351761. doi: 10.1371/journal.pone.0351761 (PMC13289875; doi:10.1371/journal.pone.0351761)
Supplement: S2 Appendix — (DOCX) [file pone.0351761.s002.docx]

# PT Navigator Role Description

Finalized Proposal

Holly Edward, PT, PhD Candidate

Supervisor: Jenna Smith-Turchyn, PT, PhD

Co-Authors:

Som Mukherjee MD, MSc, FRCP(C)

Luciana Macedo PT, PhD

Sarah Wojkowski PT, PhD

Alexander Grant, PT, CLT

Alicia Page, PT

Note: Thank you to all survey participants who contributed their valuable feedback and time to this project.

# Table of Contents

Page

#### PT Navigator Role Description

Process …………………………………………………………………………………………. 3

Assessment …………………………………………………………………………………….. 4

Triaging Decisions ……………………………………………………………………………... 5

Table 1. Outcome Measures and Decision-Making Process …………………….... 6

Main Duties and Responsibilities …………………………………………………………….. 9

Figure ………………………………………………………………………………………….. 11

References ……………………………………………………………………………………. 12

Appendix 1: Tailored Interaction Examples by Cancer Type …………………………...... 15

Appendix 2: Community Mapping …………………………………………………………. 20

### Process

- All individuals recently diagnosed with cancer (any type, any stage) who give consent and would like to meet with the PT Navigator will be referred to the PT Navigator
- Within the first month of starting adjuvant or neo-adjuvant treatment (e.g., chemotherapy, radiation, immunotherapy), the PT Navigator will perform a subjective and objective assessment (lasting approximately 1 hour) and administer evidence-based self-reported outcome measures validated for use in oncological populations
- Following the assessment, the PT Navigator will use their clinical reasoning and outcome measure scoring interpretations to guide decision making on next steps
- The PT Navigator will follow up with the patient every two to three weeks for a total of six sessions
  - To increase accessibility, sessions with the PT Navigator will be scheduled when the patient is already coming to the cancer centre for a scheduled treatment and/or appointment with their oncologist
- The PT Navigator will be notified that the patient has arrived following their check-in with the oncology care team and will receive the patient from the waiting room
- Check-in appointments (approximately 30-minutes long) will include a verbal screen for complications, repeat assessments as indicated, and consider triaging decisions based on their findings and clinical reasoning

Note: For purposes of a pilot trial, oncologists will approach their patients for interest in participating in the study. There will also be the opportunity for self-referral as recruitment flyers will be advertised within the cancer centre for patients to contact the study team directly.

### Assessment

- - Subjective: will include an assessment of current symptoms, history of present illness, medical history, social history, physical activity history, review of medications, and other pertinent information
  - Objective: will include tests such as range of motion using goniometry, grip strength, functional tests (30-second sit-to-stand, timed up and go), and aerobic testing (6-minute walk test)
  - Outcome Measures:
    - Edmonton Symptom Assessment Scale (ESAS)
    - Godin-Leisure Time Questionnaire
    - Functional Assessment of Cancer Therapy – General (FACT-G)
    - Patient-Specific Functional Scale (PSFS)

Note: The PT Navigator will use their clinical training and reasoning to personalize the assessment given each patient they see. The tests listed above are proposed as a standardized set of measures to use but the PT Navigator will not be limited to only those tests. For example, if an individual is living with breast cancer, the PT Navigator can add a lymphedema screening and relevant assessments to their follow up (see Appendix 1).

### Triaging Decisions

- The PT Navigator will move forward with one of the following steps based on their assessment:
  - 1) Develop a rehabilitation plan (i.e., discuss self-management strategies, exercise prescription, goal setting, rehabilitation barrier identification)
  - 2) Refer the individual to a community program (i.e., the patient needs more frequent follow-up and/or more supervision with rehabilitation services)
  - 3) Refer to another health care discipline (i.e., the patient needs care outside of the PT scope of practice – for example, to discuss changes in weight and appetite with a dietician)
  - 4) Continue with ongoing surveillance (i.e., no complications of treatment identified – continue to provide positive reassurance, answer questions, and provide education on maintenance strategies)

Note: Any triaging decisions made by the PT Navigator will be made with the individual’s consent and collaboration. For example, if the PT Navigator identifies that the individual requires additional physiotherapy follow up, the PT Navigator will work with the individual and their oncologist to prescribe and refer to a community clinic and/or a funded community program.

- Below is an accompanying guide created to support triaging based on outcome-assessment scores:

##### Table 1. Outcome Measures and Decision-Making Process

| **Outcome Measure** | **Outcome(s) Assessed** | **Scoring Interpretation** | **Decision-Making Process** |
| --- | --- | --- | --- |
| Edmonton Symptom Assessment Scale (ESAS)^1–4^ | 10 symptoms:  -pain  -fatigue  -nausea  -anxiety  -depression  -drowsiness  -sleep  -appetite  -well-being  -shortness of breath  -other (ex. constipation) | 0 = none  1–3 = mild  4–6 = moderate  7–10 = severe^2^  -symptoms are assessed on a 0 to 10 scale with 10 representing worst severity  -minimal clinically important difference* = 2 points^4^ | -**mild** = PT Navigator to provide education on monitoring and how to prevent symptom worsening  -**moderate/severe** = PT Navigator continues monitoring, communicate symptoms with medical team, and prescribe/deliver intervention or referral as appropriate |
| Godin Leisure-Time Questionnaire ^5–7^ | -amount of leisure-time physical activity | 24 units or more = active  14-23 units = moderately active  less than 14 units = insufficiently active/sedentary  -minimal clinically important difference* = 10% change in physical activity from baseline^7^ | -**active** = PT Navigator provides support and encouragement  -**moderately active** = PT Navigator provides physical activity education and goal setting  -**sedentary** = PT Navigator identifies barriers with patient, goal setting, and prescribe/deliver exercise intervention |
| Functional Assessment of Cancer Therapy – General  (Fact-G)^8–10^ | 27 items examining four domains of health-related quality of life (QOL):  -physical  -social  -emotional  -functional well-being | Total score: 0-108  -physical (0-28)  -social (0-28)  -emotional (0-24)  -functional (0-28)  -items are assessed from 0 to 4 with higher scores indicating better perceived QOL  -minimal clinically important difference* = 4-7 points^10^ | -**total score >90** = PT Navigator continues monitoring and providing support and education where appropriate  -**total score 50-89** = PT Navigator continues monitoring, communicate concerns with medical team, and prescribe/deliver intervention or referral as appropriate  -**total score <49** = PT Navigator informs medical team immediately for follow up, prescribe/deliver intervention or referral as appropriate |
| Patient-Specific Functional Scale (PSFS)^11^ | -functional ability | - score of 0 indicates the activity cannot be performed, while a score of 10 indicates the activity can be performed at the same level as before their problem^11^  -minimal clinically important difference not established in cancer | -a decrease in 2 points^11^ in an activity score – the PT Navigator to prescribe/deliver an exercise intervention or refer to community physiotherapy as appropriate (i.e., the patient needs frequent follow up) |
| Goniometry^12^ and Grip Strength (Dynamometer)^13–15^ | -range of motion  -muscle strength | -range of motion normative values vary per joint being examined^12^  -to compare grip strength to normative values of the general population^13^ and if applicable, normative values of patients with advanced cancer^14,15^  -minimal clinically important difference not established in cancer | -a **decrease of 10 degrees**^16^ in range of motion and/or if grip strength is **less than the normative value** for the patient’s age group and/or **there is a reduction of 5kg since baseline**^17^ - the PT Navigator to prescribe/deliver an exercise intervention or refer to community physiotherapy as appropriate (i.e., the patient needs frequent follow up) |
| 30-Second Sit to Stand^18,19^ | -functional lower extremity strength | -to compare total repetitions completed to normative values of the general population with respect to patient’s age group^18,19^  -minimal clinically important difference not established in cancer | -if score is **less than the normative value** for the patient’s age group and/or there is a **decrease in 2 repetitions since baseline**^20^ - the PT Navigator will prescribe/deliver an exercise intervention or refer to community physiotherapy as appropriate (i.e., the patient needs frequent follow up) |
| Timed Up and Go^21,22^ | -mobility, balance, and fall risk | -to compare time to normative values of the general population with respect to patient’s age group^21,22^  -minimal clinically important difference not established in cancer | -if score is **less than the normative value** for the patient’s age group and/or there is **an increase in 3 seconds since baseline**^23,24^ - the PT Navigator will prescribe/deliver an exercise intervention or refer to community physiotherapy as appropriate (i.e., the patient needs frequent follow up) |
| 6-Minute Walk Test^25,26^ | -aerobic capacity | -to compare distance walked to normative values of the general population with respect to patient’s age group^25,26^  -minimal clinically important difference* = 22-66 metres^27,28^ | -if walked distance is **less than the normative value** for the patient’s age group and/or there is a **decrease in 42 metres since baseline**^27^ - the PT Navigator will prescribe/deliver an exercise intervention or refer to community physiotherapy as appropriate (i.e., the patient needs frequent follow up) |

*Minimal clinically important difference (MCID) is the minimum difference that the patient can recognize as beneficial.^29^

**Note: this table serves as a reference guide. The PT Navigator can administer other outcome measures as appropriate and will always follow their clinical training and reasoning when considering decision-making.

### Main Duties and Responsibilities

*Initial Contact, Assessment and Early Treatment Planning (Informational and Management Continuity*^30^*):*

- Serve as the early point of contact regarding rehabilitation for persons recently diagnosed with cancer and their families/support systems
- Pro-actively manage incoming referrals to facilitate early intervention
- Gather information, conduct relevant assessments, and collaborate with individuals living with cancer to identify current and future needs, goals and risks
- Develop goals and action plans and/or recommend appropriate referrals for additional or ongoing support (i.e., social work, dietician, community programs)

*Navigation and Care Coordination (Relational Continuity*^30^*):*

- Pro-actively facilitate referrals, coordination, and communication between patients and their cancer care providers on an ongoing and regular basis
- Collaborate with the cancer care team to engage in problem solving and developing strategies to address/overcome barriers to promote patient-centred care
- Maintain positive working relationships with physicians, nurses, other allied health care professionals, and support service providers (e.g. primary care physicians, mental health, long-term care, community programs) through proactive outreach
- Routinely attend rounds and multidisciplinary team meetings as appropriate

*Follow Up and Routine Monitoring/Evaluation (Cancer Self-Management and Supportive Care*^30^*):*

- Provide ongoing support to individuals living with cancer and their families/support systems as they transition throughout their cancer journey from diagnosis to survivorship or palliative care
- Conduct routine assessments to monitor and identify early impairments if they occur
- Support positive health behaviour change through education and goal setting (i.e., physical activity, exercise)
- Continue to develop action plans and/or facilitate timely and appropriate referrals with specialist services
- Prescribe and deliver physiotherapy treatment (i.e., exercise, education, manual therapy, mobility aids, modalities)

*Standards and Quality Improvement:*

- Collect quantitative and qualitative data to support role evaluation
- Maintain comprehensive, accurate, and up-to-date chart records, in accordance with professional standards and policies
- Comply with safety responsibilities and corresponding hospital policies and procedures to facilitate a safe environment for all patients and staff
- Maintain an up-to-date knowledge of research findings and clinical practice guidelines relating to oncology physiotherapy and exercise
- Lead multi-disciplinary clinical in-service training on the role of physiotherapy and a physiotherapy navigator in oncology settings
- Participate in regular Continuing Professional Development (CPD) courses and activities

### Figure

**Figure 1. PT Navigator Role Process**


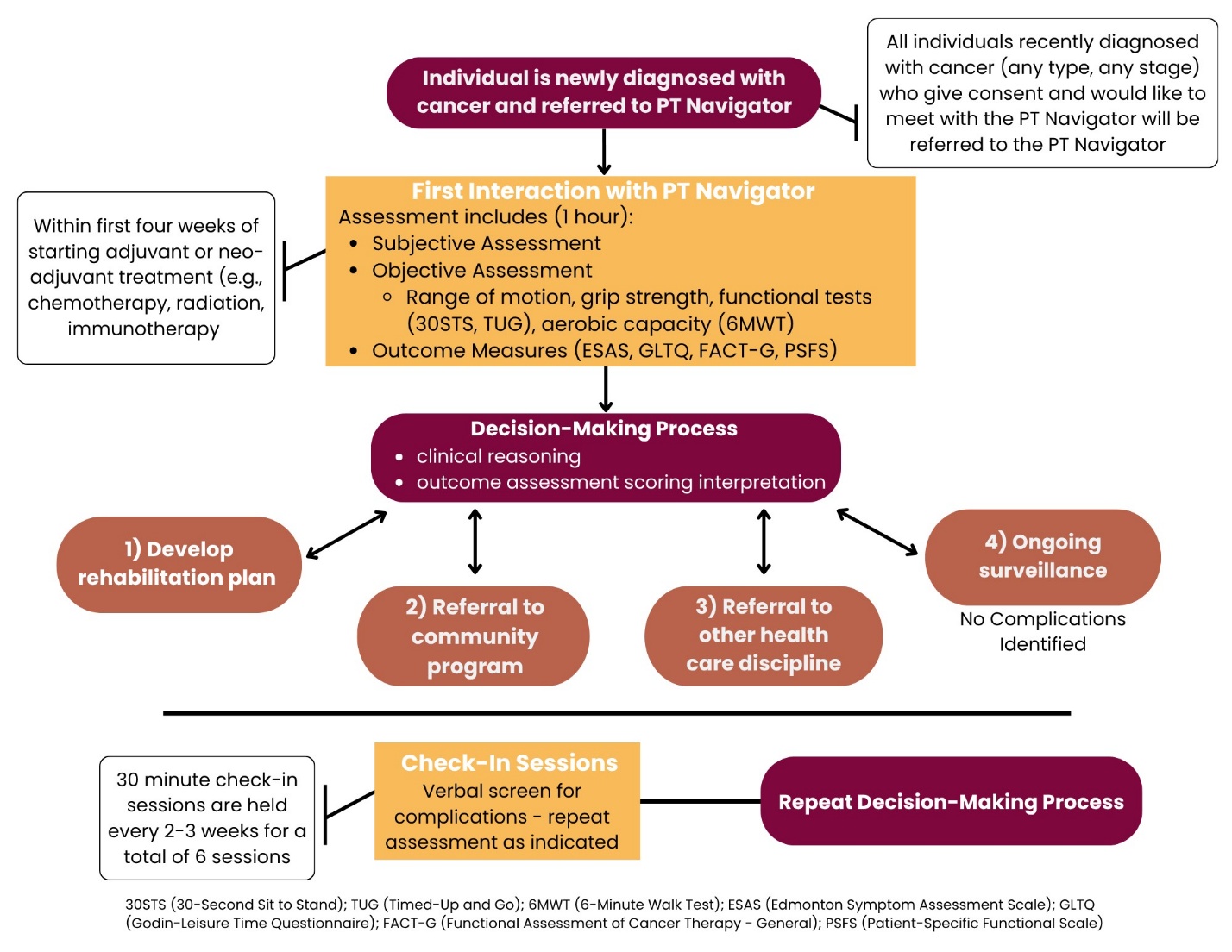


### References

1. Bruera E, Kuehn N, Miller MJ, Selmser P, Macmillan K. The Edmonton Symptom Assessment System (ESAS): a simple method for the assessment of palliative care patients. *J Palliat Care*. 1991;7(2):6-9.

2. Seow H, Sussman J, Martelli-Reid L, Pond G, Bainbridge D. Do High Symptom Scores Trigger Clinical Actions? An Audit After Implementing Electronic Symptom Screening. *J Oncol Pract*. 2012;8(6):e142-e148. doi:10.1200/JOP.2011.000525

3. Hui D, Bruera E. The Edmonton Symptom Assessment System 25 Years Later: Past, Present and Future Developments. *J Pain Symptom Manage*. 2017;53(3):630-643. doi:10.1016/j.jpainsymman.2016.10.370

4. Alberta Health Services. Edmonton Symptom Assessment System – Revised (ESAS-r) Adminstration Manual. Published online 2019. Accessed October 11, 2024. https://www.albertahealthservices.ca/assets/info/peolc/if-peolc-ed-esasr-admin-manual.pdf

5. Godin G, Shephard RJ. A simple method to assess exercise behavior in the community. *Can J Appl Sport Sci J Can Sci Appl Au Sport*. 1985;10(3):141-146.

6. Godin G. The Godin-Shephard Leisure-Time Physical Activity Questionnaire. *Health Fit J Can*. 2011;4(1):18-22. doi:10.14288/hfjc.v4i1.82

7. McNeely Ml, Sellar C, Williamson T, et al. Community-based exercise for health promotion and secondary cancer prevention in Canada: protocol for a hybrid effectiveness-implementation study. *BMJ Open*. 2019;9(9). doi:10.1136/bmjopen-2019-029975

8. P WF, A S. Psychometric analysis of the Functional Assessment of Cancer Therapy-General (FACT-G) scale in a rural sample. *Cancer*. 1997;79(12). Accessed October 11, 2024. https://pubmed.ncbi.nlm.nih.gov/9191537/

9. Brucker PS, Yost K, Cashy J, Webster K, Cella D. General population and cancer patient norms for the Functional Assessment of Cancer Therapy-General (FACT-G). *Eval Health Prof*. 2005;28(2):192-211. doi:10.1177/0163278705275341

10. Yost KJ, Eton DT. Combining distribution- and anchor-based approaches to determine minimally important differences: the FACIT experience. *Eval Health Prof*. 2005;28(2):172-191. doi:10.1177/0163278705275340

11. Stratford P. Assessing Disability and Change on Individual Patients: A Report of a Patient Specific Measure. *Physiother Can*. 1995;47(4):258-263. doi:10.3138/ptc.47.4.258

12. Norkin CC, White DJ. Normative Range of Motion Values. In: *Measurement of Joint Motion: A Guide to Goniometry, 4e*. McGraw-Hill Education; 2009. Accessed October 11, 2024. fadavispt.mhmedical.com/content.aspx?aid=1137673274

13. Wong SL. Grip strength reference values for Canadians aged 6 to 79: Canadian Health Measures Survey, 2007 to 2013. *Health Rep*. 2016;27(10):3-10.

14. Hadzibegovic S, Porthun J, Lena A, et al. Hand grip strength in patients with advanced cancer: A prospective study. *J Cachexia Sarcopenia Muscle*. 2023;14(4):1682-1694. doi:10.1002/jcsm.13248

15. Wiegert EVM, da Silva NF, de Oliveira LC, Calixto-Lima L. Reference values for handgrip strength and their association with survival in patients with incurable cancer. *Eur J Clin Nutr*. 2022;76(1):93-102. doi:10.1038/s41430-021-00921-6

16. Scalzitti DA, White DJ. Validity and Reliability of Goniometric Measurement. In: Norkin CC, White DJ, eds. *Measurement of Joint Motion: A Guide to Goniometry, 5e*. McGraw-Hill Education; 2016. Accessed March 28, 2025. fadavispt.mhmedical.com/content.aspx?aid=1186282158

17. Bohannon RW. Minimal clinically important difference for grip strength: a systematic review. *J Phys Ther Sci*. 2019;31(1):75-78. doi:10.1589/jpts.31.75

18. McKay MJ, Baldwin JN, Ferreira P, et al. Reference values for developing responsive functional outcome measures across the lifespan. *Neurology*. 2017;88(16):1512-1519. doi:10.1212/WNL.0000000000003847

19. Lein DH, Alotaibi M, Almutairi M, Singh H. Normative Reference Values and Validity for the 30-Second Chair-Stand Test in Healthy Young Adults. *Int J Sports Phys Ther*. 17(5):907-914.

20. Dunston ER, Oza S, Bai Y, et al. Preliminary Effectiveness of a Telehealth-Delivered Exercise Program in Older Adults Living With and Beyond Cancer: Retrospective Study. *JMIR Cancer*. 2025;11:e56718. doi:10.2196/56718

21. Kear BM, Guck TP, McGaha AL. Timed Up and Go (TUG) Test: Normative Reference Values for Ages 20 to 59 Years and Relationships With Physical and Mental Health Risk Factors. *J Prim Care Community Health*. 2017;8(1):9-13. doi:10.1177/2150131916659282

22. Bohannon RW. Reference Values for the Timed Up and Go Test: A Descriptive Meta-Analysis. *J Geriatr Phys Ther*. 2006;29(2):64.

23. Gautschi OP, Stienen MN, Corniola MV, et al. Assessment of the Minimum Clinically Important Difference in the Timed Up and Go Test After Surgery for Lumbar Degenerative Disc Disease. *Neurosurgery*. 2017;80(3):380-385. doi:10.1227/NEU.0000000000001320

24. Huang SL, Hsieh CL, Wu RM, Tai CH, Lin CH, Lu WS. Minimal detectable change of the timed “up & go” test and the dynamic gait index in people with Parkinson disease. *Phys Ther*. 2011;91(1):114-121. doi:10.2522/ptj.20090126

25. ATS Committee on Proficiency Standards for Clinical Pulmonary Function Laboratories. ATS statement: guidelines for the six-minute walk test. *Am J Respir Crit Care Med*. 2002;166(1):111-117. doi:10.1164/ajrccm.166.1.at1102

26. Salbach NM, O’Brien KK, Brooks D, et al. Reference values for standardized tests of walking speed and distance: A systematic review. *Gait Posture*. 2015;41(2):341-360. doi:10.1016/j.gaitpost.2014.10.002

27. Cantarero-Villanueva I, Postigo-Martin ,Paula, Granger ,Catherine L., Waterland ,Jamie, Galiano-Castillo ,Noelia, and Denehy L. The minimal clinically important difference in the treadmill six-minute walk test in active women with breast cancer during and after oncological treatments. *Disabil Rehabil*. 2023;45(5):871-878. doi:10.1080/09638288.2022.2043461

28. Granger CL, Holland AE, Gordon IR, Denehy L. Minimal important difference of the 6-minute walk distance in lung cancer. *Chron Respir Dis*. 2015;12(2):146-154. doi:10.1177/1479972315575715

29. Dettori JR, Norvell DC, Chapman JR. Clinically Important Difference: 4 Tips Toward a Better Understanding. *Glob Spine J*. 2022;12(6):1297-1298. doi:10.1177/21925682221092721

30. Fillion L, Cook S, Veillette AM, et al. Professional Navigation Framework: Elaboration and Validation in a Canadian Context. *Oncol Nurs Forum*. 2012;39:E58-69. doi:10.1188/12.ONF.E58-E69

### Appendix 1: Tailored Interaction Examples by Cancer Type

Note: This is an example of how the PT Navigator might tailor their interaction with patients diagnosed with different types of cancer. The following examples are not exhaustive, and the PT Navigator will always use their clinical reasoning when determining their assessment and triaging decisions.

Breast

*Subjective Assessment:*

- Lymphedema Screen
  - Swelling: onset and duration, location, severity, change over time
  - Skin Changes: tightness, redness, warmth, broken skin
  - Function: pain or stiffness, reduction in mobility, difficulty with ADLs/IADLs (e.g., getting dressed, bathing)

*Objective Assessment:*

- Lymphedema
  - Observation/Palpation: loss of bony prominences, pitting, fibrosis, decreased skin mobility, cording
  - Stemmer’s Sign
  - Circumferential Measurement
- Shoulder Assessment: range of motion, muscle strength, muscle length, glenohumeral capsular assessment
- Posture Assessment: changes to upper quadrant, guarding, position of scapula
- Scar Mobility: assess scar tissue, colour, thickness, stiffness, texture
- Breathing Assessment: respiratory rate, symmetry, chest expansion, use of accessory muscles
- Outcome Measures (e.g. Quick-DASH, UEFS, FACT-B)

*Physiotherapy Management:*

- Physical Activity: review of physical activity guidelines, goal setting and action planning
- Shoulder/Posture Exercises: range of motion, stretching, strengthening
- Breathing: diaphragmatic breathing, pursed lip breathing
- Lymphedema Management:
  - Self-Management: education (risk factors, signs and symptoms, skin care, lifestyle modifications), bandaging, self-massage
  - Referral to Certified Lymphedema Therapist

Lung

*Subjective Assessment:*

- Sputum: colour, consistency, frequency, amounts, smell, previous history
- Cough: onset and duration, dry vs productive, triggers, previous history
- Chest Pain

*Objective Assessment:*

- Vital Signs: Heart Rate, Respiratory Rate, Blood Pressure, Oxygen Saturation (SpO_2_), Temperature
- IPPA: Inspection, Palpation, Percussion, Auscultation
- Dyspnea: Borg Dyspnea Scale
- Shoulder Assessment: range of motion, muscle strength, muscle length, glenohumeral capsular assessment
- Posture Assessment: changes to upper quadrant, guarding, position of scapula
- Outcome Measures (e.g. Quick-Dash, UEFS, SGRQ, Activities of Daily Living; Katz Index, Barthel Index)

*Physiotherapy Management:*

- Respiratory Exercises: diaphragmatic breathing, splinted coughing, incentive spirometry
- Neck, Scapular and Shoulder Exercises: range of motion, stretching, strengthening
- Palliative: management of breathlessness, relaxation techniques, activity pacing, assistance with mobilization, gait-aid training, functional goal-directed exercises

**Prostate**

*Subjective Assessment:*

- Pelvic Health
  - Bladder Habits
  - Sexual Dysfunction
  - Pelvic Pain
  - Incontinence
- Lymphedema Screen
  - Swelling: onset and duration, location, severity, change over time
  - Skin Changes: tightness, redness, warmth, broken skin
  - Function: pain or stiffness, reduction in mobility, difficulty with ADLs/IADLs (e.g., getting dressed, bathing)

*Objective Assessment:*

- Pelvic Health
  - External Assessment (e.g., posture, breathing, core strength, hip/low back movement assessment)
  - Outcome Measures (e.g., Expanded Prostate Cancer Index Composite)
- Lymphedema
  - Observation/Palpation: loss of bony prominences, pitting, fibrosis, decreased skin mobility, cording
  - Stemmer’s Sign
  - Circumferential Measurement

*Physiotherapy Management:*

- Pelvic Health
  - Bladder Diary
  - Education (e.g., bladder habits, lifestyle changes)
  - Hip/Low Back/Core Exercises: range of motion, stretching, strengthening
  - Refer to Pelvic Health PT
- Lymphedema Management:
  - Self-Management: education (risk factors, signs and symptoms, skin care, lifestyle modifications), bandaging, self-massage
  - Referral to Certified Lymphedema Therapist

**Colorectal**

*Subjective Assessment:*

- Pelvic Health
  - Bowel Habits
  - Sexual Dysfunction
  - Pelvic Pain
  - Incontinence
- Stoma Functioning
- Gastro-Intestinal Symptoms
- Lymphedema Screen
  - Swelling: onset and duration, location, severity, change over time
  - Skin Changes: tightness, redness, warmth, broken skin
  - Function: pain or stiffness, reduction in mobility, difficulty with ADLs/IADLs (e.g., getting dressed, bathing)

*Objective Assessment:*

- Stoma Assessment: appearance, output, palpation, skin assessment
- Pelvic Health
  - External Assessment (e.g., posture, breathing, core strength, hip/low back movement assessment)
  - Outcome Measures (e.g., Functional Assessment of Cancer Therapy – Colorectal)
- Lymphedema
  - Observation/Palpation: loss of bony prominences, pitting, fibrosis, decreased skin mobility, cording
  - Stemmer’s Sign
  - Circumferential Measurement

*Physiotherapy Management:*

- Stoma Management: education, breathing exercises, core strengthening, lifting techniques
- Pelvic Health
  - Fibre/Bowel Diary
  - Education (e.g., bowel habits, lifestyle changes)
  - Hip/Low Back/Core Exercises: range of motion, stretching, strengthening
  - Refer to Pelvic Health PT
- Lymphedema Management:
  - Self-Management: education (risk factors, signs and symptoms, skin care, lifestyle modifications), bandaging, self-massage
  - Referral to Certified Lymphedema Therapist

Other Key Considerations:

- *Cancer-Related Fatigue*
  - Assessment: signs and symptoms, cognitive effects, impact on daily life, outcome measures (e.g., Brief Fatigue Inventory)
  - Self-Management: energy conservation, sleep hygiene, psychosocial therapy (e.g., stress management, support groups)
  - Physical Activity: review of physical activity guidelines, goal setting and action planning
  - Referral to Community Programs and/or Complementary Therapies (e.g., massage, acupuncture)
- *Chemotherapy-Induced Peripheral Neuropathy*
  - Assessment: signs and symptoms, proprioception, reflexes, sensation, gait, balance, fine motor dexterity
  - Self-Management: safety education, self massage, neuromuscular stimulation/use of TENS, prescribing gait-aids and braces (e.g. AFO), balance training
  - Consult Medical Team Regarding Pharmacological Therapies
  - Referral to OT
  - Referral to Community Programs and/or Complementary Therapies (e.g., massage, acupuncture)
- *Goals of Treatment Interventions*
  - Restorative (to pre-diagnostic levels of function)
  - Compensatory (maximize function during treatment)
  - Palliative (multidisciplinary supportive care to enhance functional independence and mobility)

### Appendix 2: Community Mapping

##### Physical Activity & Exercise

- [CanWell Program](https://www.ymcahbb.ca/health-fitness/livewell-health-management)
  - CanWell Program includes supervised exercise, group fitness classes, health professional consultation, and education. Program included in YMCA membership.
  - Financial assistance available.
  - [Physician Referral Form](https://www.ymcahbb.ca/canwell-referral-form)
- [MacWarriors Cancer Exercise Program](https://pace.mcmaster.ca/exercise-programs/exercise-program-for-adults-with-cancer/)
  - Physiotherapy-based, supervised exercise sessions for cancer survivors. Before joining group exercise sessions, a registered physiotherapist will carry out an intake assessment to help ensure safe and effective outcomes. Exercise plans are individualized based on each individual’s diagnosis, treatment, and recovery status.
  - [Physician Referral Form](https://pace.mcmaster.ca/app/uploads/2020/09/MacWarriors-Cancer-Exercise-Program-Physician-Referral-Form-January-2020.pdf)
  - Initial Assessment: $160; Membership: $50/month – $600 total (12-month commitment); $55/month – $330 total (6-month commitment); Parking: $20/month
  - Financial Assistance Available
- [YWCA Hamilton](https://www.ywcahamilton.org/encore-plus)
  - ENCOREplus is the umbrella program that includes YWCA Encore, Pilates Beyond Breast Cancer and Walk, Talk & Wander Beyond Breast Cancer programs that support women in different stages of breast cancer recovery.
  - Free exercise programs for women who have had breast cancer.
- Note: See Wellwood and Wellspring Niagara (*see Social and Emotional Support*) for additional physical activity and exercise-related programs.

##### Transportation

- [Wheels of Hope](https://cancer.ca/en/living-with-cancer/how-we-can-help/transportation)
  - Driving Program
    - To use our driving program, you must be able to get in and out of a vehicle without assistance. If you cannot travel independently, you must bring a travel escort (at least 18 years of age) with you in the vehicle to each of your appointments. Please note, we are unable to transport wheelchairs.
    - Clients need to be referred to our volunteer driver program by a member of their healthcare team at the hospital or cancer clinic where they are receiving treatment.
    - Healthcare Professionals can request a copy of our referral form by contacting [transportation@cancer.ca](mailto:transportation@cancer.ca).
  - Travel Treatment Fund
    - Our Travel Treatment Fund offers short-term financial assistance to offset some travel costs when traveling to cancer treatments. This includes expenses like fuel and taxi or public transit fares. It is available to people in Canada who are currently or will soon be undergoing cancer treatment.
    - To apply, complete and submit a [Travel Treatment Fund Application Form](https://cdn.cancer.ca/-/media/files/living-with-cancer/how-we-can-help/transportation/nationwide_ttf_application_form_en.pdf?rev=717e80af649447f0b3ff94b525961351&hash=10B02F83C6DA2C9FC7F77C66BF0F964B). (See Section 6 of the Application for information about where to send your completed form). Please also submit a copy of a "VOID" cheque or direct deposit form provided by your banking institution (if you want to receive money by direct deposit).

##### Social and Emotional Support

- [Wellwood](https://www.wellwood.ca/)
  - Community-based, non-profit organization that provides tools, information, programs, and peer support for people who have received a diagnosis of cancer, loved ones, caregivers and healthcare providers.
- [Wellspring Niagara](https://wellspring.ca/niagara/) (Fonthill)
  - Offers a wide variety of programs and services to meet the social, emotional, restorative and informational needs of people living with cancer and their caregivers. All programs are free of charge – delivered in-person and/or online.

##### Lymphedema

- [Hamilton Area Lymphedema Resources](https://www.hamiltonhealthsciences.ca/wp-content/uploads/2019/02/Hamilton-Area-Lymphedema-Resources-Mar-21-2022.pdf)
  - List of CDT and ADP Authorizers

##### Additional Resources

- [Cancer Assistance Program](https://cancerassist.ca/)
  - Home Health Equipment
    - Loans of home health equipment (e.g., walkers, wheelchairs, bathroom equipment, cushions)
  - Personal Care
    - Provides patients with a range of nutritional supplements, incontinence items, wigs and head coverings and mastectomy garments. For any questions or to book an appointment for a wig or mastectomy item fitting, or to schedule a delivery of personal care items, please contact at 905-383-9797. Visit capwigstore.ca to view our collection of wigs before booking appointment.
  - Transportation
    - Provides residents of the Greater Hamilton Area a safe ride with door-to-door service to and from all cancer-related medical appointments. With a quick phone call, patients can register for rides according to their treatment schedules with our dedicated volunteer drivers.
    - Hours of Operation: 9:00am to 4:00pm, Monday to Friday
    - When booking your drive, please be prepared to provide the following information: Location and time of appointment, Type of appointment and approximate length (if you know), If you will have someone with you on the trip and if you will have any mobility equipment
  - Parking Assistance
    - Provides limited parking spaces in a private lot at Sacred Heart Parish Centre within walking distance to Juravinski Cancer Centre. You must obtain your own personal parking permit from the CAP office and call to reserve your spot before each appointment. To book your appointment, call 905-383-9797 x105 or email info@cancerassist.ca.
- [Community Services Locator](https://csl.cancer.ca/en)
  - Offered by the Canadian Cancer Society, the online tool can help people living with cancer find services and programs such as support groups, wigs, prostheses, and more.
- [Mark Preece Family House](https://markpreecehouse.ca/)
  - The Mark Preece Family House is a caring place to call home for families of patients in Hamilton area hospital – located near Hamilton General Hospital.
  - Referral Form (Member of Heath Care Team): <https://markpreecehouse.ca/our-services/>
